# Urine biomarkers in ESSIC type 2 interstitial cystitis/bladder pain syndrome and overactive bladder with developing a novel diagnostic algorithm

**DOI:** 10.1038/s41598-020-80131-5

**Published:** 2021-01-13

**Authors:** Yuan-Hong Jiang, Jia-Fong Jhang, Yung-Hsiang Hsu, Han-Chen Ho, Ya-Hui Wu, Hann-Chorng Kuo

**Affiliations:** 1 Department of Urology, Hualien Tzu Chi Hospital, Buddhist Tzu Chi Medical Foundation and Tzu Chi University, 707, Section 3, Chung Yang Road, Hualien, Taiwan; 2 Department of Pathology, Hualien Tzu Chi Hospital, Buddhist Tzu Chi Medical Foundation and Tzu Chi University, Hualien, Taiwan; 3 Department of Anatomy, Tzu Chi University, Hualien, Taiwan

**Keywords:** Biochemistry, Urology

## Abstract

This study aimed to investigate the diagnostic values of urine cytokines in interstitial cystitis/bladder pain syndrome (IC/BPS) and overactive bladder (OAB) patients, and to develop a novel diagnostic algorithm. Urine samples were collected from 40 IC/BPS, 40 OAB patients, and 30 controls. Commercially available multiplex immunoassays were used to analyze 31 targeted cytokines. Urine cytokine profiles were significantly different among study groups and controls. MIP-1β showed the highest sensitivity (92.2%) for identifying diseased study patients from controls. The cytokines with high diagnostic values for distinguishing between IC and OAB included IL-10, RANTES, eotaxin, CXCL10, IL-12p70, NGF, IL-6, IL-17A, MCP-1, and IL-1RA. The diagnostic algorithm was subsequently developed according to the diagnostic values obtained. MIP-1β was selected for the initial screening test to diagnose diseased patients and controls with diagnostic rates of 81.6% and 68.4%, respectively. As confirmation tests for IC/BPS, the diagnostic rates of eotaxin, CXCL10, and RANTES were 73.3%, 72.7%, and 69.7%, respectively. As the confirmation test for OAB, the diagnostic rate of IL-10 was 60%. Urine cytokine profiles of IC/BPS and OAB patients differed from those of controls and might be useful as biomarkers for diagnosis. A novel pilot diagnostic algorithm was developed based on these profiles.

## Introduction

Interstitial cystitis/bladder pain syndrome (IC/BPS) is a chronic inflammatory urinary bladder disorder characterized by bladder pain and associated urinary frequency, urgency, nocturia, and sterile urine^1^. Overactive bladder (OAB) is defined as a symptom complex characterized by urinary urgency, usually accompanied by frequency and nocturia, but with or without urgency urinary incontinence, in the absence of causative infection or pathologic condition^2,3^. Overlapping symptoms of IC/BPS and OAB often make the two conditions difficult to distinguish and may complicate subsequent diagnosis and treatment algorithms^4,5^. More sensitive diagnostic tools to discriminate between IC/BPS from OAB are thus required.

Urothelial dysfunction, increased suburothelial inflammation, and apoptosis occur in many lower urinary tract diseases, including IC/BPS^6^, OAB^7^, and neurogenic voiding dysfunction^8^. Although both IC/BPS and OAB feature chronic bladder inflammation, their pathophysiologic differences are still unclear. Furthermore, IC/BPS and OAB share some overlapping symptoms and have similar histopathological characteristics and even some potential urinary biomarkers^9,10^.

The analysis of multiple urinary proteins is a convenient approach to monitoring inflammation in bladder tissue^9^. Different lower urinary tract diseases may exhibit different protein profiles and biochemical contents, reflecting distinct pathophysiologies, and intrinsic bladder conditions. IC/BPS patients have demonstrated distinct urine cytokine profiles compared with controls, which makes urine cytokines to be potential biomarkers for the diagnosis and mapping of the clinical characteristics of IC/BPS^11^. In OAB patients, urinary nerve growth factor (NGF), brain-derived neurotrophic factor, and adenosine triphosphate are increased, and the use of these biomarkers in identifying distinct OAB phenotypes is expected in the future^10^.

Currently, only few studies have investigated the urine inflammatory protein profiles between IC/BPS and OAB^12,13^. Furuta et al. reported IC/BPS patients had increased inflammatory urine markers, including vascular endothelial growth factor (VEGF) and CXCL10, than OAB patients^13^. However, the study lacked a control group, and more importantly, distinct biomarkers in the urine specimen of OAB patients were not discovered, causing limited benefits in clinical application.

This study investigated the diagnostic values of urine cytokines in IC/BPS and OAB in comparison with controls. A novel diagnostic algorithm was then developed to distinguish IC and OAB from controls using the diagnostic values obtained.

## Results

The characteristics of eligible study and controls patients are shown in Table 1. Significantly different distributions in age, gender, comorbidity of diabetes mellitus, and body mass index were observed among the groups. In the IC/BPS group, the mean O'Leary–Saint symptom score was 22.0 ± 7.0 with a mean maximal bladder capacity under anesthesia of 591.3 ± 120.9 mL. Among OAB patients, the mean overactive bladder symptom score (OABSS), and International Prostate Symptom Score were 10.1 ± 3.3, and 12.6 ± 6.2, respectively.

**Table 1:** Clinical characteristics of IC/BPS, OAB, and control patients.

	(A) OAB N = 40	(B) IC/BPS N = 40	(C) Control N = 30	P-value	Post hoc analysis
Age	64.7 ± 8.9 (39–78)	49.3 ± 12.1 (21–69)	57.7 ± 10.1 (39–71)	< 0.001	A vs B A vs C B vs C
Gender	F30, M10	F34, M6	F30	0.009	A vs C
DM	13 (33%)	2 (5%)	3 (10%)	0.002	A vs B
BMI	26.05 ± 3.49	21.94 ± 3.92	25.60 ± 4.39	< 0.001	A vs B B vs C
VAS	NA	3.7 ± 2.9	NA		
ICSI	NA	11.4 ± 4.2	NA		
ICPI	NA	10.4 ± 3.3	NA		
OSS	NA	22.0 ± 7.0	NA		
MBC (mL)	NA	591.3 ± 120.9	NA		
Glomerulation grade	NA	2.2 ± 0.4	NA		
OABSS	10.1 ± 3.3	NA	NA		
IPSS-S	8.0 ± 3.6	NA	2.7 ± 2.2	< 0.001	
IPSS-V	4.6 ± 4.5	NA	1.3 ± 1.4	< 0.001	
IPSS-T	12.6 ± 6.2	NA	4.0 ± 2.5	< 0.001	

*DM* diabetes mellitus, *BMI* Body Mass Index, *VAS* visual analogue scale pain score, *ICSI* interstitial cystitis symptom index, *ICPI* interstitial cystitis problem index, *OSS* O’Leary-Saint score, *MBC* maximal bladder capacity under anesthesia, *OABSS* overactive bladder symptoms score, *IPSS* International Prostate Symptom Score, *IPSS-S* IPSS storage subscore, *IPSS-V* IPSS voiding subscore, *IPSS-T* total IPSS score, *NA* not available.

Table 2 shows the targeted urine cytokine levels among IC/BPS, OAB, and control patients. For each targeted cytokine, the numbers of outliers ranged from 0 to 2, and was no more than 5%. Urine cytokine profiles were significantly different among the IC/BPS, OAB, and control groups. IC/BPS patients showed urine cytokine levels distinct from those of OAB patients, which included MCP-1, RANTES, eotaxin, NGF, CXCL10, IL-10, and IL-17A.

**Table 2:** Urine cytokine/chemokine levels of IC/BPS, OAB, and control groups.

Urine cytokines^b^	(A) OAB N = 40	(B) IC/BPS N = 40	(C) Control N = 30	P-value	Post hoc analysis
MCP-1	240.83 ± 188.74 (2)	524.61 ± 511.31 (0)	147.14 ± 109.74 (1)	0.000	A vs B A vs C B vs C
MIP-1^α^^a^	1.60 ± 1.03 (0)	1.09 ± 0.58 (0)	1.34 ± 0.75 (1)	0.021	A vs C
MIP-1β	2.91 ± 2.27 (1)	2.93 ± 1.38 (2)	2.52 ± 1.82 (1)	0.614	
RANTES	6.65 ± 5.23 (1)	12.75 ± 7.59 (1)	6.04 ± 5.15 (1)	0.000	A vs B B vs C
Eotaxin	4.65 ± 3.73 (2)	11.87 ± 8.41 (1)	4.98 ± 3.70 (0)	0.000	A vs B B vs C
G-CSF	7.92 ± 7.58 (1)	7.04 ± 7.05 (2)	11.72 ± 10.17 (1)	0.071	
GM-CSF^a^	1.35 ± 0.48 (2)	1.30 ± 0.46 (0)	1.24 ± 0.50 (1)	0.653	
VEGF^a^	13.64 ± 4.95 (1)	12.82 ± 6.68 (1)	10.97 ± 5.00 (0)	0.149	
NGF	0.27 ± 0.07 (1)	0.35 ± 0.15 (1)	0.26 ± 0.08 (0)	0.002	A vs B B vs C
EGF	5454.83 ± 3767.41 (0)	6833.88 ± 4476.67 (0)	6224.35 ± 4906.68 (0)	0.370	
CXCL10	24.77 ± 41.77 (1)	62.24 ± 49.15 (2)	13.81 ± 18.43 (1)	0.000	A vs B B vs C
IFNα2	3.66 ± 1.75 (1)	3.39 ± 1.89 (1)	3.22 ± 1.52 (1)	0.568	
IFNγ	1.23 ± 0.19 (2)	1.19 ± 0.30 (0)	1.19 ± 0.19 (1)	0.783	
TNFα	0.82 ± 0.34 (1)	0.71 ± 0.25 (1)	0.82 ± 0.33 (1)	0.219	
TNFβ^a^	0.79 ± 0.13 (1)	0.74 ± 0.12 (1)	0.76 ± 0.12 (1)	0.210	
IL-1^α^^a^	1.80 ± 1.39 (0)	1.35 ± 0.44 (1)	1.43 ± 0.75 (1)	0.089	
IL-1β^a^	0.50 ± 0.15 (2)	0.52 ± 0.16 (1)	0.56 ± 0.27 (1)	0.445	
IL-1RA	390.56 ± 507.03 (1)	467.29 ± 396.94 (1)	325.52 ± 387.05 (1)	0.414	
IL-2^a^	0.72 ± 0.17 (0)	0.82 ± 0.17 (0)	0.80 ± 0.19 (1)	0.028	A vs B
IL-3^a^	0.59 ± 0.22 (0)	0.51 ± 0.17 (0)	0.64 ± 0.25 (0)	0.034	B vs C
IL-4	15.3 ± 10.88 (1)	12.44 ± 8.57 (0)	11.07 ± 15.16 (1)	0.293	
IL-5^a^	0.61 ± 0.23 (1)	0.44 ± 0.11 (1)	0.54 ± 0.17 (1)	0.000	A vs B B vs C
IL-6	1.73 ± 2.34 (1)	2.36 ± 3.18 (2)	1.29 ± 1.35 (1)	0.214	
IL-7	1.41 ± 0.44 (1)	1.59 ± 0.63 (1)	1.52 ± 0.81 (1)	0.424	
IL-8	10.83 ± 12.28 (2)	10.97 ± 11.06 (0)	12.45 ± 20.98 (1)	0.890	
IL-10	1.48 ± 0.45 (1)	0.97 ± 0.31 (1)	1.23 ± 0.32 (1)	0.000	A vs B A vs C B vs C
IL-12p40^a^	0.90 ± 0.40 (1)	0.99 ± 0.45 (0)	0.79 ± 0.32 (0)	0.106	
IL-12P70	1.34 ± 0.33 (1)	1.14 ± 0.38 (1)	1.28 ± 0.42 (0)	0.063	
IL-13^a^	1.19 ± 0.38 (1)	1.21 ± 0.29 (1)	1.24 ± 0.40 (1)	0.841	
IL-15	1.64 ± 0.91 (1)	1.43 ± 0.55 (1)	1.21 ± 0.37 (1)	0.029	A vs C
IL-17A	0.93 ± 0.18 (1)	0.80 ± 0.17 (0)	0.97 ± 0.20 (1)	0.000	A vs B B vs C

() indicating the number of outliers.

^a^The values in study groups below the minimum detectable concentrations according to the assay manufacturer.

^b^Units: pg/mL.

**Figure 1:**
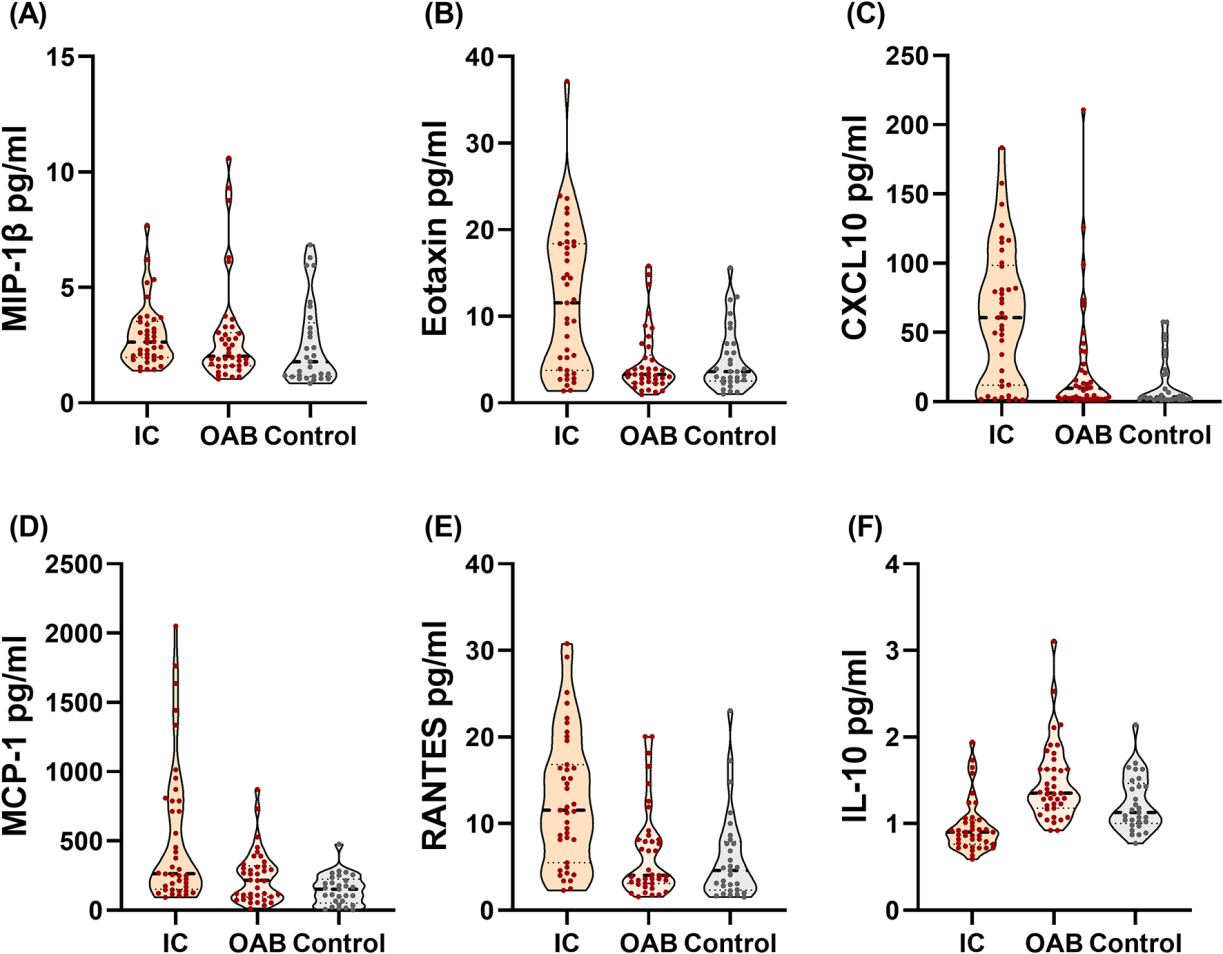
Violin plots of significant cytokines with high diagnostic values for IC/BPS and OAB.

Table 3 summarizes the diagnostic values of each urine cytokine. MIP-1β was the cytokine with the highest sensitivity (92.2%) to discriminate diseased study patients from controls with the optimal cut-off value of 1.385 pg/mL. The cytokines with high diagnostic values (areas under the receiver operating characteristic curves > 0.7) to distinguish IC and OAB included IL-10, RANTES, eotaxin, CXCL10, IL-12p70, NGF, IL-6, IL-17A, MCP-1, and IL-1RA. Figure 1 shows violin plots of selected significant cytokines with high diagnostic values.

**Table 3:** Diagnostic values of urine cytokines between diseased patients (IC/BPS and OAB) and controls.

Urine cytokines	AUC	Cut-off value^c^:	Sensitivity (%)	Specificity (%)	PPV (%)	NPV (%)
**Diagnostic values between diseased patients (IC/BPS and OAB) and controls**						
MCP-1	0.753	204.235	60.3	72.4	85.5	40.4
IL-4	0.703	10.610	54.4	86.2	91.5	41.0
CXCL10	0.685	7.500	66.2	65.5	83.6	42.2
MIP-1β	0.674	1.385	92.2	44.8	81.6	68.4
RANTES	0.666	7.805	53.8	75.9	85.7	37.9
IL-8	0.651	2.785	82.1	44.8	80.0	48.1
IL-17A^a^	0.642	0.925	65.8	62.1	82.5	40.0
IL-6	0.631	1.165	50	79.3	86.7	37.7
NGF	0.624	0.315	41.0	80.0	84.2	34.3
IL-15	0.611	0.855	97.4	24.1	77.6	77.8
G-CSF	0.605	4.820	53.2	72.4	83.7	36.8
Eotaxin	0.604	7.270	40.3	80.0	83.8	34.3
**Diagnostic values between IC/BPS and OAB**						
IL-10^b^	0.829	1.025	74.4	92.3	90.6	78.3
RANTES	0.814	9.305	61.5	82.1	77.4	68.1
Eotaxin	0.774	9.035	59.0	89.5	85.2	68.0
CXCL10	0.768	40.495	65.8	82.1	78.1	71.1
IL-12p70^b^	0.739	1.085	51.3	94.9	90.9	66.1
NGF	0.725	0.355	35.9	84.6	70.0	56.9
IL-6	0.716	1.515	52.6	82.1	74.1	64.0
IL-17A^b^	0.708	0.885	70.0	59.0	63.6	65.7
MCP-1	0.704	120.385	90.0	42.1	62.1	80.0
IL-1RA	0.703	113.05	89.7	43.6	61.4	81.0

*AUC* area under the curve of ROC (receiver operating characteristic), *PPV* positive predictive value, *NPV* negative predictive value.

^a^Higher values of targeted cytokines indicated the diagnosis of controls.

^b^Higher values of targeted cytokines indicated the diagnosis of OAB.

^c^Units: pg/mL.

Controlling for age, gender, body mass index, and the comorbidity of diabetes mellitus, multivariate logistic regression models revealed the odds ratio (OR) of the diagnostic value of targeted cytokines (Table 4). The urine cytokines that differentiated OAB patients from controls included MCP-1 (OR 1.692) and IL-10 (OR 1.288), and those that differentiated IC/BPS patients from controls included MCP-1 (OR 2.569), CXCL10 (OR 1.516), eotaxin (OR 1.181), RANTES (OR 1.15), and NGF (OR 1.102). Moreover, many urine cytokines were found to vary between IC/BPS and OAB, which demonstrates potential in distinguishing different urinary disease statuses and controls.

**Table 4:** Multivariate models (controlling for age, gender, BMI, and DM).

	P value	Odds ratio	95% CI	Odds ratio units*
**OAB vs. control**				
MCP-1	0.032	1.692	1.046–2.734	100
IL-10	0.004	1.288	1.083–1.531	0.1
**IC/BPS vs. control**				
MCP-1	0.008	2.569	1.282–5.148	100
CXCL10	0.004	1.516	1.146–2.005	10
Eotaxin	0.003	1.181	1.058–1.319	1
RANTES	0.014	1.15	1.028–1.286	1
NGF	0.027	1.102	1.011–1.200	0.01
**IC/BPS vs.OAB**				
MCP-1	0.006	1.358	1.092–1.688	100
Eotaxin	0.001	1.294	1.115–1.503	1
CXCL10	0.005	1.246	1.068–1.454	10
RANTES	0.004	1.201	1.060–1.361	1
NGF	0.028	1.126	1.013– 1.252	0.01
IL-17A	0.007	0.939	0.897–0.983	0.01
IL-12p70	0.003	0.678	0.522–0.879	0.1
IL-10	0.001	0.633	0.484–0.828	0.1
IFNα2	0.008	0.520	0.322–0.840	1

*Units: pg/mL.

**Figure 2:**
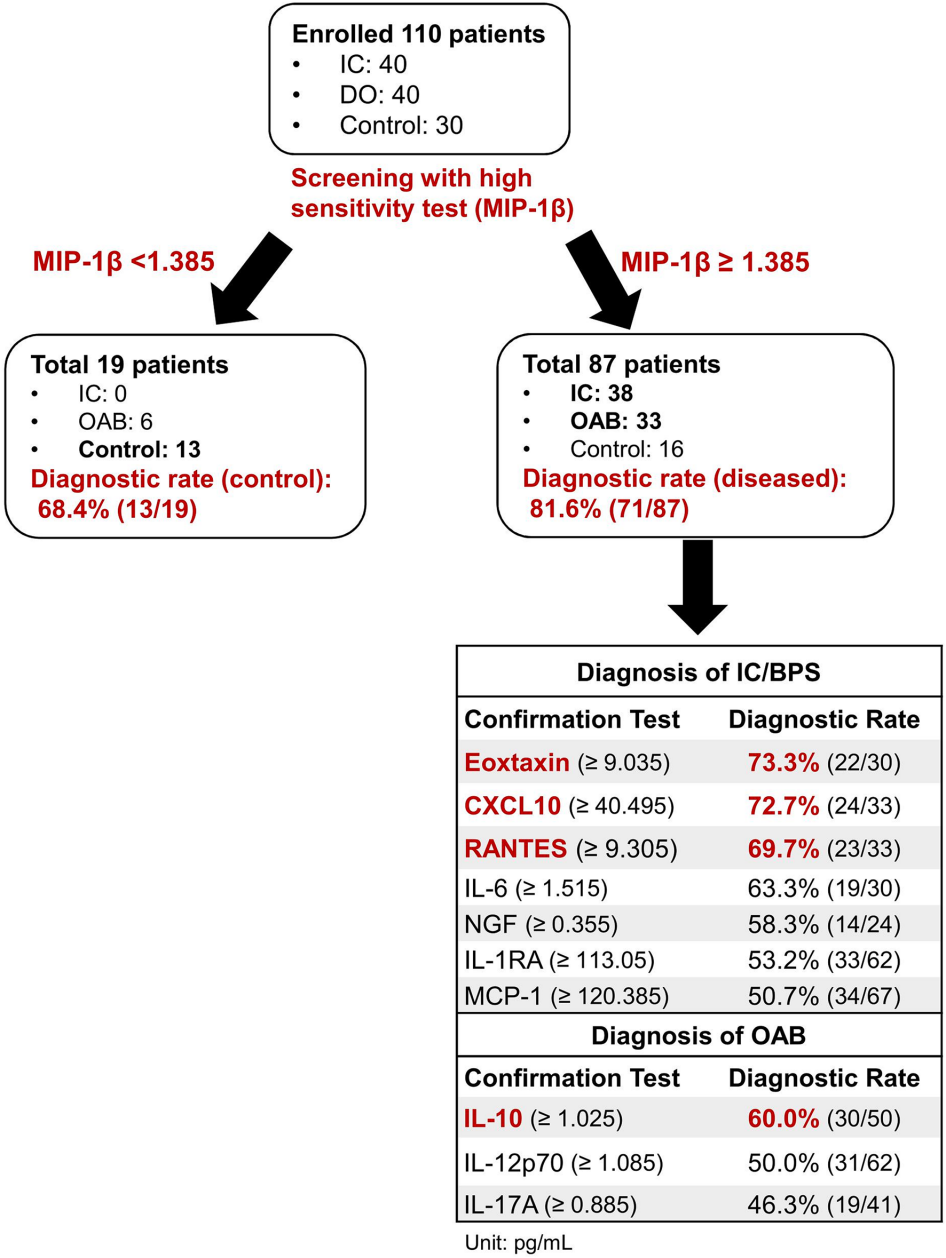
Preliminary novel diagnostic algorithm for distinguishing IC/BPS and OAB from controls based on diagnostic values.

We proposed a pilot diagnostic algorithm using the diagnostic values of obtained (Fig. 2). MIP-1β, with the highest sensitivity, was selected as the initial screening test to diagnose diseased patients from controls, with diagnostic rates of 81.6% and 68.4%, respectively. Next, the cytokines with high diagnostic values for distinguishing between IC/BPS and OAB were set as the confirmation tests. As confirmation tests for IC/BPS, the diagnostic rates of eotaxin, CXCL10, and RANTES were 73.3%, 72.7%, and 69.7%, respectively, whereas, as the confirmation test for OAB, the diagnostic rate of IL-10 was 60%.

## Discussion

Both IC/BPS and OAB patients presented different urine cytokine profiles compared with controls, which might reflect their distinct pathologic conditions in the bladder. This study assessed the diagnostic values of each urine cytokine, conducted multivariate model analysis for controlling confounding factors, and proposed a pilot diagnostic algorithm to identify IC/BPS and OAB patients from controls based on the results. This novel diagnostic algorithm might be applied clinically to distinguish among conditions with similar storage-related lower urinary tract symptoms. This non-invasive approach to urine cytokine analysis also provides important information regarding the pathological bladder conditions in patients with IC/BPS and OAB. Urine cytokines have important roles in the diagnosis of IC/BPS and OAB with the potential to serve as novel biomarkers.

MIP-1β, also known as chemokine ligand 4, is a member of the C–C chemokine family and a chemoattractant for natural killer cells, monocytes, and a variety of other immune cells^14^. MIP-1β was reportedly elevated in urine samples from OAB^15^ and IC/BPS^11^ patients and was presumed to be related to bladder inflammation. However, the specific roles of MIP-1β in the pathogenesis of OAB and IC/BPS have been unclear. In this study, MIP-1β was the cytokine with the highest sensitivity (92.2%) to discriminate diseased study patients from controls with the optimal cut-off value of 1.385 pg/mL. Elevated MIP-1β levels might be the common characteristic of urine specimen in both IC/ BPS and OAB patients. Accordingly, MIP-1β was selected as the initial screening test to diagnose diseased patients from controls in this pilot diagnostic algorithm with the diagnostic rate of 81.6%. Elevated MIP-1β levels could be a common biochemical indicator for both IC/BPS and OAB.

MCP-1, CXCL10, and RANTES are known to be upregulated and involved in chemokine signaling in peripheral neuroinflammatory responses^16^. These cytokines were elevated in IC/BPS urine specimens, suggesting neuropathic inflammation, as well as afferent hypersensitivity within the bladder^11^. Niimi et al. reported that urine CXCL10 was not increased in 25 non-Hunner type IC/BPS patients in compared with 31 controls^17^. However, in our previous study with 127 non-Hunner type IC/ BPS patients and 28 controls, urine CXCL10 levels were not only significantly increased in IC/BPS patients but also correlated with the glomerulation grade and maximal bladder capacity^11^. Eotaxin-1, acting as a selective chemoattractant for eosinophils, is implicated in many eosinophilic inflammatory diseases^18^. Elevated eotaxin levels in IC/BPS urine specimens suggest an autoimmune response and allergy-related inflammation in IC/BPS^1,11^. In the present study, these cytokines were increased in IC/BPS patients and remained statistically significant in differentiating IC/BPS from OAB in multivariate logistic regression models. These findings suggest that MCP-1, CXCL10, and RANTES could be crucial in IC/BPS but not in OAB. Therefore, these cytokines were selected as the confirmation tests of IC/BPS in the pilot diagnostic algorithm, and the diagnostic rates of eotaxin, CXCL10, and RANTES were 73.3%, 72.7%, and 69.7%, respectively. The high diagnostic rates indicate the promising clinical application of these cytokines as biomarkers of IC/BPS.

IL-10, a cytokine with anti-inflammatory properties, has a crucial role in preventing inflammatory and autoimmune pathologies^19^. Together with pro-inflammatory cytokines, IL-10 is also induced in many situations and affects the development of an immune response. In one small-scale case study, OAB patients showed increased urine IL-10 levels compared with controls^15^. It pointed out the possible mechanisms for elevated urine IL-10 levels, including inflammation induced by sterile trauma^20^ and the compensatory response to tissue inflammation^21^. In the present study, urine IL-10 levels were increased in OAB but not in IC/BPS patients as compared with controls. In addition, IL-10 was the analyte, which could significantly differentiate OAB from IC/BPS, as demonstrated by multivariate logistic regression analysis. Accordingly, IL-10 was selected as the confirmation test for OAB in this pilot diagnostic algorithm.

Urothelial dysfunction, the activation of C-fibers and release of substance P, and neurogenic inflammation with mast cell activation are the core pathophysiology of IC/BPS^22^. Neurotrophic factors and neurogenic inflammation associated with bladder afferent hyperexcitability pathways are the featured inflammatory mechanisms in IC/BPS. Akiyama et al. reported that Hunner type IC/BPS patients had a distinct gene expression profile of bladder mucosal tissue, but the genetic profile did not differ between the other types of IC/BPS and controls^23^. It indicated that Hunner type IC/BPS had a distinct underlying pathophysiology; however, the evidence in the other types of IC/BPS could not be conclusive due to the small sample size. In OAB, suburothelial inflammation with mast cell infiltration was also explored^7^, with non-specifically increased neurotrophic factors in urine^10^. IC/BPS and OAB, although two dissimilar diseases, shared overlapping symptoms^24^, similar histopathological characteristics^6,7^, and similar potential urinary biomarkers^9,10^. The research on the distinct pathophysiologic differences between IC/BPS and OAB still continues. Figure 2 shows that MIP-1β levels increased in both the IC/BPS and OAB groups; MCP-1, CXCL10, RANTES, and eotaxin increased in the IC/BPS group; and IL-10 increased in the OAB group. Some of the urine analytes were non-specifically elevated in both IC/BPS and OAB, but some were specific to the respective groups, indicating their common and distinct inflammation-related pathomechanisms, respectively.

There were several limitations in this study. First, the case numbers in the study groups were small. Enrolling more patients in future investigations is thus needed. Second, most study patients and all controls were women, which may have created bias with the gender effect. Third, the possibility of intra-individual variation and other systemic inflammatory diseases and comorbidities also may have confounded the urine cytokine levels. Fourth, the lack of prospective symptom assessment with the same questionnaires in OAB and IC/BPS patients. Finally, we excluded the outliers of the urine cytokines levels for further analysis, although the percentage of outliers was small. Currently, there is no ideal single test to differentiate OAB and IC/BPS, and to use a panel of biomarkers might elevate the diagnostic rates and help to diagnose these diseases earlier. However, the calculation and analysis of the combination of biomarkers needs a larger case number. In the future, to conduct a more comprehensive and more well-designed study with more case number is needed.

## Conclusion

The urine cytokine profiles of IC/BPS and OAB patients markedly differed from those of controls, indicating urine cytokines as potential biomarkers for diagnosing IC/BPS and OAB. Based on the findings, a novel pilot diagnostic algorithm was developed with promising clinical applications.

## Materials and methods

### Patients

From June 2016 to April 2019, we prospectively enrolled 40 IC/BPS patients, and 40 medical refractory OAB patients at the Department of Urology of Hualien Tzu Chi Hospital, Taiwan. All enrolled patients were Taiwanese Asian.

The diagnostic criteria for IC/BPS, based on the proposed guidelines of the European Society for the Study of Interstitial Cystitis, constituted “chronic pelvic pain, pressure, or discomfort perceived to be related to the urinary bladder accompanied by at least one other urinary symptom, such as persistent urge to void or urinary frequency, for more than 6 months,” and the exclusion of potentially similar diseases^11,25^. All enrolled IC/BPS patients were European Society for the Study of Interstitial Cystitis type 2 (i.e. with glomerulations detected during hydrodistention). The diagnosis of OAB was made clinically using OABSS questionnaires with OABSS $\ge$ 3. All enrolled OAB patients were refractory to medical treatment for 3 months and confirmed to have urodynamic detrusor overactivity, but without other voiding dysfunction in video-urodynamic studies. Exclusion criteria of enrolled patients included active urinary tract infection, neurogenic voiding dysfunction (including multiple sclerosis, spinal cord injury, cerebrovascular accidents, and Parkinson’s disease), a history of bladder surgery/or traumatic injury, a history of urethral or prostate surgery, a history of urinary tract malignancy or tuberculosis, a history of pelvic radiation, a history of nephrotic or nephritic syndrome, urolithiasis, and/or impaired renal function (serum creatinine > 2.0 mg/dL).

Additionally, 30 women with genuine stress urinary incontinence who were ready to undergo anti-incontinence sling surgeries served as controls. All controls did not have other significant lower urinary tract symptoms (defined as International Prostate Symptom Score < 6), or other storage or voiding dysfunction in video-urodynamic studies.

### Clinical investigation

In IC/BPS patients, the assessment of clinical symptoms included the O'Leary–Saint symptom score, interstitial cystitis symptom index, interstitial cystitis problem index, and visual analog scale pain score. We also recorded findings on cystoscopic hydrodistention, including maximal bladder capacity under anesthesia, and the grade of glomerulations. In OAB patients, the assessment of clinical symptoms included OABSS and the International Prostate Symptom Score.

### Urine biomarkers investigation

Urine samples were collected from all enrolled study patients and controls before the surgical procedures. Urine was self-voided when the subjects reported a full bladder sensation. Urinalysis was performed simultaneously to confirm an infection-free status before urine samples were stored. The preparation of urine samples, the analytes used for investigation in urine samples, and the laboratory procedures were similar to those in our previous study^11^. Inflammation-related cytokines, chemokines, and neurotrophins in urine samples were assayed using commercially available microspheres with the Milliplex Human cytokine/chemokine magnetic bead-based panel kit (Millipore, Darmstadt, Germany). A total of 31 targeted analytes were used for the multiplex kit, including inflammatory cytokines and chemokines: catalog number HCYTMAG-60K-PX30 (EGF, eotaxin, G-CSF, GM-CSF, IFNα2, IFNγ, IL-10, IL-12p40, IL-12p70, IL-13, IL-15, IL-17A, IL-1RA, 1L-1α, IL-1β, IL-2, IL-3, IL-4, IL-5, IL-6, IL-7, IL-8, CXCL10, MCP-1, MIP-1α, MIP-1β, RANTES, TNFα, TNFβ, and VEGF), and catalog number HADK2MAG-61K (NGF). The following laboratory procedures of the quantification of targeted analytes were performed similarly to those in our previous study^11^. All the laboratory procedures were approved by the department of medical research of Hualien Tzu Chi Hospital, and were performed in accordance with relevant guidelines and regulations.

This study was approved by the Institutional Review Board and Ethics Committee of Buddhist Tzu Chi General Hospital (No. IRB107-175-A). All study patients and controls were informed of the rationale and procedures of this study, and written informed consent was obtained from each participant.

### Statistical analysis

Continuous variables are represented as means ± standard deviations, and categorical data are represented as numbers and percentages. For each targeted cytokine, values outside the range between means ± 3 standard deviations in each study or control group were defined as outliers and excluded from further analysis. Differences in clinical data and urine cytokine levels were analyzed using ANOVA, and post hoc analysis was performed. Cytokines with mean values below the minimum detectable concentrations as per the assay manufacturer were excluded for further analysis.

Receiver operating characteristic curves were generated for the diagnostic value of each cytokine, and areas under the receiver operating characteristic curves were calculated. Multivariate logistic regression models for controlling the confounding factors were fit for each analyte, and OR was calculated. All calculations were performed using SPSS Statistics for Windows, Version 20.0 (IBM Corp., Armonk, NY, USA). Differences were considered statistically significant if p values were less than 0.05.
